# The anti-tumor effect of Apo2L/TRAIL on patient pancreatic adenocarcinomas grown as xenografts in SCID mice

**DOI:** 10.1186/1479-5876-3-22

**Published:** 2005-05-19

**Authors:** Bonnie L Hylander, Rose Pitoniak, Remedios B Penetrante, John F Gibbs, Dilek Oktay, Jinrong Cheng, Elizabeth A Repasky

**Affiliations:** 1 Department of Immunology, Roswell Park Cancer Institute, Buffalo, NY, 14263, USA; 2 Department of Pathology, Roswell Park Cancer Institute, Buffalo, NY, 14263, USA; 3 Department of Surgery, Roswell Park Cancer Institute, Buffalo, NY, 14263, USA; 4 Department of Experimental Therapeutics, Roswell Park Cancer Institute, Buffalo, NY, 14263, USA

## Abstract

**Background:**

Apo2L/TRAIL has considerable promise for cancer therapy based on the fact that this member of the tumor necrosis factor family induces apoptosis in the majority of malignant cells, while normal cells are more resistant. Furthermore, in many cells, when Apo2L/TRAIL is combined with chemotherapy, the effect is synergistic. The majority of this work has been carried out using cell lines. Therefore, investigation of how patient tumors respond to Apo2L/TRAIL can validate and/or complement information obtained from cell lines and prove valuable in the design of future clinical trials.

**Methods:**

We have investigated the Apo2L/TRAIL sensitivity of patient derived pancreatic tumors using a patient tumor xenograft/ SCID mouse model. Mice bearing engrafted tumors were treated with Apo2L/TRAIL, gemcitabine or a combination of both therapies.

**Results:**

Patient tumors grown as xenografts exhibited a spectrum of sensitivity to Apo2L/TRAIL. Both Apo2L/TRAIL sensitive and resistant pancreatic tumors were found, as well as tumors that showed heterogeneity of response. Changes in apoptotic signaling molecules in a sensitive tumor were analyzed by Western blot following Apo2L/TRAIL treatment; loss of procaspase 8, Bid and procaspase 3 was observed and correlated with inhibition of tumor growth. However, in a tumor that was highly resistant to killing by Apo2L/TRAIL, although there was a partial loss of procaspase 8 and Bid in response to Apo2L/TRAIL treatment, loss of procaspase 3 was negligible. This resistant tumor also expressed a high level of the anti-apoptotic molecule Bcl-X_L _that, in comparison, was not detected in a sensitive tumor. Importantly, in the majority of these tumors, addition of gemcitabine to Apo2L/TRAIL resulted in a greater anti-tumor effect than either therapy used alone.

**Conclusion:**

These data suggest that in a clinical setting we will see heterogeneity in the response of patients' tumors to Apo2L/TRAIL, including tumors that are highly sensitive as well as those that are resistant. While much more work is needed to understand the molecular basis for this heterogeneity, it is very encouraging, that Apo2L/TRAIL in combination with gemcitabine increased therapeutic efficacy in almost every case and therefore may be a highly effective strategy for controlling human pancreatic cancer validating and expanding upon what has been reported for cell lines.

## Introduction

The high mortality rate seen in patients with pancreatic cancer reflects both the difficulty in early detection and the lack of effective treatment to augment surgery [1,2] so that, following diagnosis, the average survival time of the majority of patients is between 4–5 months [3]. Within the last few years, the use of the deoxycytodine analog gemcitabine has been shown to result in improved clinical benefit, slightly longer mean survival time and has become the first line chemotherapy for pancreatic adenocarcinoma [4,5]. However, since the five-year survival rate has remained at 4%, many new approaches to the treatment of pancreatic adenocarcinoma are being investigated [5,6]. Several of these approaches focus on combination therapies in which gemicitabine is combined with a second cytotoxic agent (e.g. auristatin-PE, [7]), or a targeted biological therapy (e.g.; the anti-EGFR antibody C225, [8,9]; OSI-774, Tarceva, [10]).

In 1995, a new member of the tumor necrosis factor (TNF) family was independently identified by two different groups and named TRAIL (Tumor Necrosis Factor Related Apoptosis Inducing Ligand, [11]) and Apo2L (based on its homology to Fas/Apo1L [12]). This molecule induces apoptosis in a large number of human tumor cell lines, both *in vitro *and *in vivo*, while normal cells are not susceptible [11-15]. This is in contrast to other members of this family of ligands (i.e. TNF and FasL), which have marked toxicity when administered systemically (for further discussion see recent reviews by [16-18]). An important natural role for Apo2L/TRAIL in the immunosurveillance of tumors has been proposed based on its expression on several immune cells, including activated NK and T cells (see [19]for discussion). This natural role of Apo2L/TRAIL in anti-tumor activity provides further rationale for attempting to develop Apo2L/TRAIL as a therapeutic molecule. The original studies with Apo2L showed that it could act synergistically with the chemotherapeutic agents 5-FU and CPT-11 in animal studies using a colon tumor cell line [14,20]. There have since been numerous studies expanding these observations using a large number of cell lines of different tumor types with a variety of chemotherapies, both *in vitro *and *in vivo*. Among the types of solid tumors that have been studied are breast [21], lung [22], prostate [23], mesothelioma, [24], renal [25], ovarian [26], bladder [27], glioma [28] and pancreas [29]. However, there is a concern that these results might not be predictive of the response of actual patients' tumors. Therefore, an investigation of how patient tumors respond to Apo2L/TRAIL could validate and/or complement information obtained from cell lines and prove valuable in the design of future clinical trials.

Our group has previously investigated the efficacy of Apo2L/TRAIL and CPT-11 combination therapy on *patient-derived *colon tumors [30] using a SCID mouse xenograft model that our lab has developed [31-34]. The value of this model is that it enables evaluation of actual patient tumors that retain the heterogeneity and histological architecture of the original tumor. TRAIL exerted a significant anti-tumor effect on three different patient colon tumors grown as xenografts and this effect was significantly augmented by the addition of CPT-11 or 5-FU [30]. However, use of this model has also revealed the existence of patient-derived colon tumors which are resistant to Apo2L/TRAIL alone but are sensitive to the combination of Apo2L/TRAIL and CPT-11 (Kenji, manuscript in preparation) This suggested the possibility that a differential response to Apo2L/TRAIL may occur between patients and that additional research is critical for 1) appreciating the degree to which variability occurs between tumors, 2) developing strategies for using Apo2L/TRAIL in combination therapies and 3) determining methods for predicting ahead of time which patients will benefit from Apo2L/TRAIL.

It has previously been reported that pancreatic cell lines exhibit varying degrees of sensitivity to Apo2L/TRAIL and that some of these lines are extremely resistant [35,29,37]. It has also been reported that resistant cells can be sensitized to Apo2L/TRAIL (e.g. [29,38]). However, although the combination of Apo2L/TRAIL and gemcitabine *in vitro *has been investigated using pancreatic cell lines, there have been conflicting reports on whether this combination does [39] or does not [40] have a synergistic cytotoxic effect.

In this paper, we describe our experience in evaluating five different patient pancreatic tumors grown in SCID mice to Apo2L/TRAIL. The recombinant form of human Apo2L/TRAIL used shows low activity against the murine TRAIL receptor and therefore this model may not reveal any potential toxicities of Apo2L/TRAIL, however it does provide a relevant model for evaluating the sensitivity of patient tumors. Our data support the idea that some patients' tumors may exhibit significant sensitivity while others may be resistant. Still other patients' tumors may be heterogeneous and exhibit regions of both sensitivity and resistance. However, the combination of Apo2L/TRAIL and gemcitabine can enhance the anti-tumor effect against Apo2L/TRAIL sensitive tumors and, importantly, can overcome resistance to either single agent, and result in suppression of resistant tumors. Thus, these findings predict that patients' tumors will exhibit both sensitivity and resistance to Apo2L/TRAIL treatment and that it may be possible to develop approaches for overcoming this resistance by combining Apo2L/TRAIL and chemotherapy.

## Materials and methods

### Patient pancreatic tumor-SCID mouse model

Our use of the SCID mouse-patient tumor xenograft model has been previously described ([31,33,41,32,34,30]). For these studies, surgical specimens of patients' pancreatic tumors were received shortly after resection through the Tissue Procurement Facility (TP) of RPCI and cut into 2 mm × 2 mm pieces in tissue culture medium (RPMI 1640) under sterile conditions. SCID mice were then anesthetized by intraperitoneal injection of 0.4–0.5 ml Avertin (2.5 g 2,2,2-tribromoethanol dissolved in 5 ml 2-methyl-butanol/200 ml ddH_2_O) and individual tumor pieces were implanted subcutaneously in the abdominal wall of three mice (1^st ^passage) and monitored for growth. The mice used in all experiments were 7–8 weeks old CB17 SCID mice with an average weight of 18–20 g. They were kept in sterile cages (4–5 mice per cage) and fed with autoclaved chow and water. Mice were maintained in air-conditioned and light controlled rooms (12 hr cycles). All procedures, injections and tumor measurements were carried out under a laminar flow hood using aseptic precautions. Tumor specimens that grew to a size of 1 cm (8–12 weeks) were retrieved and subsequently passaged into recipient mice (2^nd ^passage) and were considered to have successfully engrafted when these tumors grew. Pathological diagnosis of patient specimens and evaluation of engrafted/ passaged tumors was performed in collaboration with a member of the Pathology Department at RPCI.

### Experimental design

Five different pancreatic adenocarcinomas that successfully engrafted into SCID mice were selected for passage into groups of experimental mice. Tumors reached 4–5 mm in diameter in approximately 4–6 weeks and the mice were divided into experimental groups of similar tumor sizes. These tumors are referred to as Tumor #1 (TP#10791), Tumor #2 (TP#10978), Tumor #3 (TP#11424), Tumor #4 (TP#12424), and Tumor #5 (TP#11727).

### Apo2L/TRAIL

Apo2L/TRAIL used in this investigation was prepared by Genentech, Inc. as described previously [14] and provided as a gift by Genentech and Amgen. A cycle of treatment with Apo2L/TRAIL consisted of daily intraperitoneal injection of 500 μg Apo2L/TRAIL /200 ul saline for 14 days. Mice received 2 such cycles separated by a 7–10 day rest period. Control mice received sterile saline. Tumor volume was calculated with the formula V= LD × (SD)^2 ^/ 2, where V is the tumor volume, LD is the longest tumor diameter and SD is the shortest tumor diameter. Data was graphed and the Students unpaired t-test was calculated using SigmaPlot. At various time points during and at the termination of an experiment, mice were sacrificed and pieces of tumor were fixed in formalin, snap-frozen in cryovials in liquid nitrogen, or both, for subsequent analysis. Sections of all tumor samples were processed for light microscopy by standard methods.

### Chemotherapy

Gemcitabine (Gemzar, Eli Lilly obtained from McKesson, Buffalo, NY) was administered by intraperitoneal injection daily 5 days/wk in two two-week cycles with a rest interval of one week at doses between 1.0 – 2.5 mg/kg as indicated. Therefore mice received 7.5–13.5 mg/kg weekly, which is less than that routinely administered clinically to patients (25 mg/kg/week).

### TUNEL assay

Apoptosis was evaluated by terminal deoxynucleotidyl transferase-mediated dUTP-nick end-labeling (TUNEL) staining (ApopTag, Intergen, Corp) according to the manufacturers instructions.

### Western blotting

Cell and tissue lysates (lysis buffer containing 20 mM Tris pH 7.5, 120 mM NaCl, 100 mM NaF, 0.5% Nonionic P_40, _200 μM Na_3_VO_4, _50 mM β-Glycerophosphate, 10 mM NaPPi, 4 mM PMSF, 10 μg/ml Leupeptine, 2 mM Benzamidine, 10 μg/ml Aprotinin) were separated by SDS-PAGE and transferred to nitrocellulose membrane. Blots were immunostained by standard techniques: non-specific binding was blocked and membranes were incubated overnight at 4°C with primary antibody. Antibodies used were anti-caspase 8 (Oncogene # AM46), anti-Bcl-X_L, _(Cell Signaling #2762), anti-Bid (Cell Signaling #2002), anti-human mitochondria (Chemicon #Mab 1273; recognizes a 65 kd epitope on the membrane of intact human mitochondria) and anti-Caspase 3 (Imgenex IMG-144). This was followed by washing, incubation with peroxidase-conjugated secondary antibody and visualization of the bands by enhanced chemoluminescence (ECL) and exposure of the blots to Kodak BioMax film. Anti β-actin was used as a loading control.

### Immunohistochemistry

Immunohistochemical evaluation of p53 (mouse monoclonal antibody DO-7, Novocasta) was performed on sections of formalin fixed, paraffin embedded tumors. Antigen retrieval was accomplished with DAKO Target Retrieval Solution using a Black and Decker steamer for 20 minutes followed by a 20 minute cooling period.

## Results and discussion

### Patient pancreatic adenocarcinomas engrafted into SCID mice maintain the histological features of the original tumor

**Figure 1:**
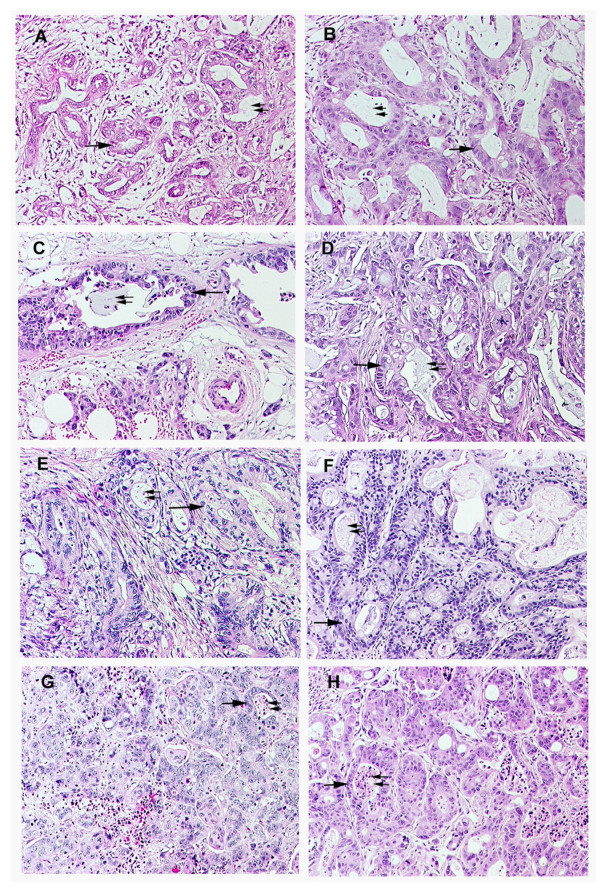
The histological features of patient pancreatic adenocarcinomas (left hand panels: **A, C, E, G**) are maintained when these tumors are grown as xenografts (right hand panels: **B, D, F, H**) in SCID mice. The well differentiated glands (arrows) containing secretory material (double arrows) which are seen in surgical specimens, proliferate in xenografts of the same tumors. (Tumor #1: A, B; Tumor #2: C, D; Tumor #3: E, F; Tumor #4: G, H). Original magnification, ×10.

In order to evaluate the sensitivity of patient pancreatic tumors to novel therapeutic agents, we have developed a patient tumor/SCID mouse xenograft model in which specimens of pancreatic adenocarcinomas obtained directly from surgeries performed at Roswell Park Cancer Institute are established as xenografts in SCID mice. Successful engraftment has been obtained in 33% of pancreatic tumors (18 of 53 tumors implanted over a six year period), including both adenocarcinomas and neuroendocrine tumors. The tumors used in this study were selected randomly from available patient tumor xenografts. Histological evaluation of these tumors demonstrated that engrafted tumors maintained a remarkable degree of similarity, in terms of the histological features seen, to the original tumors. The malignant cells of the xenografts formed glands resembling those in the patients' tumors and secretory material was often seen in the lumen of these glands (Figure 1). As in the patient tumors, these glands are separated and surrounded by stromal tissue.

### The growth of patient-derived pancreatic adenocarcinomas can be inhibited by Apo2L/TRAIL

**Figure 2:**
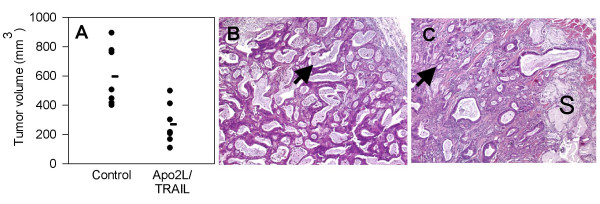
Apo2L/TRAIL significantly inhibited the growth of pancreatic adenocarcinoma #1. Mice bearing patient tumor xenografts were treated with Apo2L/TRAIL and the tumor volumes on the final day of treatment (day 35) were compared (7 mice/group, the mean is indicated by the horizontal line; p = 0.004). B, C: Comparison of the histology of the median tumors. B. In the periphery of an untreated tumor, there are numerous large glands (arrow) containing secretory product. C. The treated tumor has fewer glands (arrow) and a higher proportion of connective tissue. There are also large pools of residual secretory product (S) that are not surrounded by epithelial cells.

**Figure 3:**
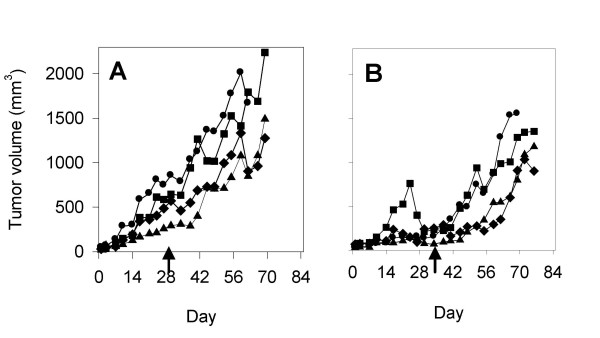
Growth of Tumor #2 is also significantly inhibited during treatment with Apo2L/TRAIL. **A**. Control; **B. **Apo2L/TRAIL treated. (4 mice/group; day 35, p = 0.016). Following cessation of treatment (arrows), growth of treated tumors progressed at a rate similar to that of the untreated tumors.

In our initial experiment, we treated mice engrafted with Tumor #1 with two cycles of Apo2L/TRAIL following a 5 week schedule (500 μg/mouse- daily for 2 weeks, 1 week no treatment, daily for 2 weeks). At the end of the five weeks, the sizes of the tumors in the treated group were significantly smaller that those in the control group (Figure 2A). Histological analysis of the median tumor in each group showed that there were areas of substantial necrosis in the interior of these tumors. However, the periphery of an untreated tumor consisted of numerous glands of fairly uniform size containing copious amounts of secretory material (Figure 2B). In contrast, in the smaller, treated tumor (Figure 2C), the majority of the remaining glands were smaller, further apart and contained less secretory material. Also, in comparison to the untreated tumor, the treated tumor consisted of proportionately more connective tissue. Interestingly, in the periphery of the treated tumor, there were pools of secretory material that were not contained in glands, suggesting that the epithelial cells that formed these glands had been lost (Figure 2C). However, even though there was a marked anti-tumor effect of Apo2L/TRAIL on this tumor, the tumor was not totally eradicated and areas of viable tumor cells remained. Similar results were obtained with a second patient tumor (Tumor #2; Figure 3). The growth of this tumor was also significantly inhibited during the 35 day schedule of treatment with Apo2L/TRAIL. However, we observed that tumor growth resumed after cessation of treatment and these tumors grew at a rate similar to that of the untreated controls. Therefore, although Apo2L/TRAIL can significantly suppress tumor growth, it may not prove to be totally efficacious as a single agent and it is important to develop strategies to use it in combination with other therapeutic agents.

### Combination therapy with Apo2L/TRAIL and gemcitabine resulted in enhanced inhibition of tumor growth over that of either single agent alone

**Figure 4:**
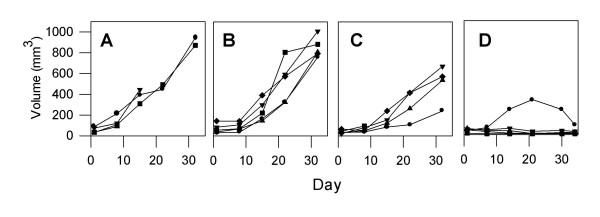
Evaluation of the response of Tumor #2 to increasing doses of gemcitabine. **A**. Control; **B**. Tumors in mice treated with 1.0 mg/kg gemcitabine grow at a rate comparable to that of untreated tumors; **C**. Tumors in mice treated with 1.5 mg/kg gemicitabine show growth inhibition to varying degrees (p = .045); **D**. Tumors in mice treated with 2.5 mg/kg gemcitabine alone show uniform suppression (p < .001).

We next carried out a series of experiments to determine whether the efficacy of Apo2L/TRAIL against patient derived pancreatic adenocarcinomas could be enhanced by using it in combination with gemicitabine. The results of an experiment using Tumor #2, demonstrated a dose dependent response of this tumor to gemcitabine and based on the fact that 1.5 mg/kg moderately, but incompletely, suppressed tumor growth (Figure 4), we chose this dose for future experiments in order to best observe any benefit of combination therapy. Subsequently, tumor bearing mice were treated with 500 μg of Apo2L/TRAIL alone (for a 35 day schedule as above), 1.5 mg/kg of gemcitabine alone (5x/week) or the two agents in combination. The responses of four different patient tumors to combination therapy were investigated and the results indicate interesting diversity in the responses of these tumors.

**Figure 5:**
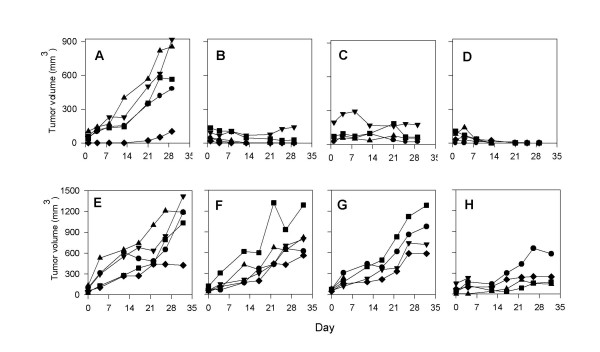
The effect of Apo2L/TRAIL in combination with gemcitabine on an Apo2L/TRAIL sensitive and resistant pancreatic adenocarcinoma. **A-D**. Tumor #3; **E-H**. Tumor #4. Groups of tumor bearing mice were treated with: **A&E**- Controls, **B&F**- Apo2L/TRAIL alone, **C&G**- gemcitabine alone, **D&H**- Apo2L/TRAIL and gemcitabine in combination. Tumor #3 is sensitive to Apo2L/TRAIL alone (B; p = 0.006) while Tumor #4 is resistant to Apo2L/TRAIL (F). This differential sensitivity is also seen in the response to gemcitabine alone (C, p = 0.02, and G). The efficacy of Apo2L/TRAIL in combination with gemcitabine appears to be enhanced in Tumor #3 (D) and is significantly greater than either treatment alone in Tumor #4 (H; p = 0.008).

One tumor, Tumor #3 was sensitive to both Apo2L/TRAIL and gemcitabine alone and underwent significant growth inhibition in response to either of these agents. Although growth suppression by combination treatment was not statistically different from the effects of either single treatment alone, the anti-tumor effect appears to be enhanced and these tumors underwent complete regression (Figure 5, A–D). This tumor was consistently sensitive to Apo2L/TRAIL in several passages (data not shown).

In contrast to Tumor #3, Tumor #4 exhibited resistance to both Apo2L/TRAIL and gemcitabine when administered as single agents. However, the use of these two agents in combination was able to overcome this resistance and significant tumor growth inhibition was achieved (Fig 5, E–H ).

**Figure 6:**
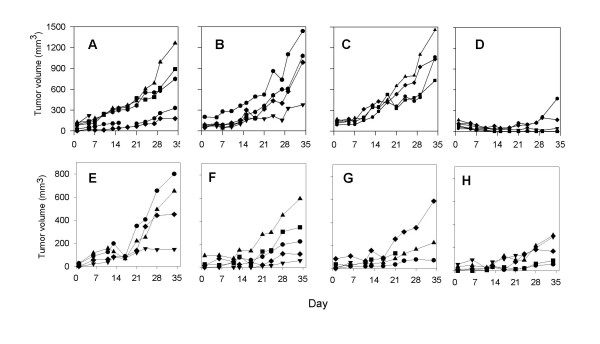
The anti-tumor effect of Apo2L/TRAIL and gemcitabine in combination is greater than that of either single agent alone. Mice were engrafted with two different patient tumors: **A-D**, Tumor #2; **E-H**, Tumor #5. Treatment: A, E. Control; B, F. Apo2L/TRAIL; C, G. gemcitabine; D, H. combination therapy. Although tumors showed variable degrees of sensitivity to either Apo2L/TRAIL or gemcitabine alone, the combination treatment resulted in significant inhibition of tumor growth (D, p = 0.035; H, p = 0.05).

**Figure 7:**
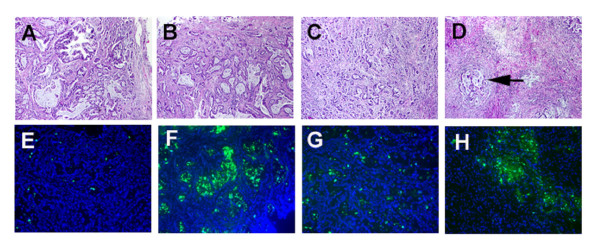
The effect of Apo2L/TRAIL and gemcitabine alone and in combination (Tumor #2, day 5 of treatment). **A**. Many large glands are apparent in an untreated tumor. **B, C**. Tumors treated with Apo2L/TRAIL (B) or gemicitabine (C) contain fewer, smaller glands. **D**. Tumors treated with a combination of Apo2L/TRAIL and gemcitabine consist mainly of fibrotic tissue with foci of residual tumor cells (D, arrow). **E-H**. TUNEL assay. Few apoptotic cells are apparent in either the untreated (**E**) or gemcitabine treated (**G**) treated tumors. The Apo2L/TRAIL (**F**) and combination treated (**H**) tumors contain numerous cells undergoing apoptosis.

Tumor #2 showed variability in sensitivity to Apo2L/TRAIL. Although, when originally evaluated, Tumor #2 had demonstrated sensitivity to Apo2L/TRAIL alone, in a subsequent experiment, growth was not significantly suppressed by either Apo2L/TRAIL or gemcitabine alone (Figure 6, A–D). In this case, however, tumor growth was significantly inhibited by treatment with the agents in combination. During this experiment, one representative tumor was removed from each group on day 7 and prepared for histology (Figure 7, A–D). The normal histological appearance of this adenocarcinoma is shown in a control; this tumor consisted of elaborate glands containing copious secretory material and a moderate amount of stroma (Figure 7A). In comparison, both the Apo2L/TRAIL (Figure 7B) and gemcitabine (Figure 7C) treated tumors demonstrated evident histological changes with fewer, smaller glands. In marked contrast to the tumors treated with single agents alone, the tumor treated with the combination therapy consisted almost entirely of fibrotic connective tissue and contained only isolated foci of viable tumor cells (Figure 7D). Staining of these tumors with the TUNEL assay showed very few apoptotic cells in the untreated or gemicitabine alone treated tumors (Figure 7, E and 7 G). However, large numbers of apoptotic cells are present in both the tumors treated with Apo2L/TRAIL alone and Apo2L/TRAIL in combination with gemcitabine (Figure 7, F and 7 H). This experiment with Tumor #2 was repeated and although there was heterogeneity in the growth of tumors within each treatment group, the results were similar in that significant tumor growth inhibition was achieved by combination therapy (data not shown). The evaluation of Tumor #5 (Figure 6, E–H) yielded similar results in that growth of the tumors in the untreated group was variable and tumor growth was not significantly suppressed by either Apo2L/TRAIL or gemcitabine. However, combination therapy was able to significantly suppress growth of Tumor #5 (Figure 6H).

At this time, the basis for the cooperation between Apo2L/TRAIL and gemcitabine is not known. It is likely that knowledge about the expression of molecules such as p53 in these tumors will be important in understanding factors which affect sensitivity to chemotherapy. Although we have not sequenced p53 for mutations, we have evaluated p53 expression by immunohistochemistry in Tumors #2, 3, 4 and 5. Whereas p53 overexpression was not detected in Tumors #2, 3 and 4, heterogeneous overexpression was detected in Tumor #5 (data not shown). This suggests that the responses of these tumors to combination therapy may be independent of p53 status. It has been demonstrated that the response of tumors to gemcitabine can occur in a p53 independent manner in [42]. Future studies investigating the mechanism(s) of cooperation between Apo2L/TRAIL and gemicitabine will include analyses of the status of critical molecules such as p53.

Thus, these tumors demonstrated a heterogeneous range of responses to Apo2L/TRAIL. Tumor #3 is significantly sensitive to killing with Apo2L/TRAIL alone and this was consistently seen in subsequent experiments. Interestingly, Tumors #4 and 5 were resistant to Apo2L/TRAIL alone in the passage that was evaluated. It is informative that with Tumor #2, which was evaluated in several experiments, the response varied in different passages. Although the basis for this variability is unknown, it seems likely that this is the result of an inherent heterogeneity in the original tumor. Alternatively, this may indicate the response of this tumor to different factors in the tumor microenvironment. Interestingly, even when Tumor #2 was not inhibited by Apo2L/TRAIL alone, an increased amount of apoptosis was detected within the tumor early in the treatment. Although this variability is problematic, it is likely reflective of the situation that can be expected in the clinic and therefore, it is especially encouraging that combination therapy with gemcitabine shows such potential for complementing and enhancing the anti-tumor effect of Apo2L/TRAIL in the majority of these tumors.

### Investigation of the basis for the difference in sensitivity to Apo2L/TRAIL

**Figure 8:**
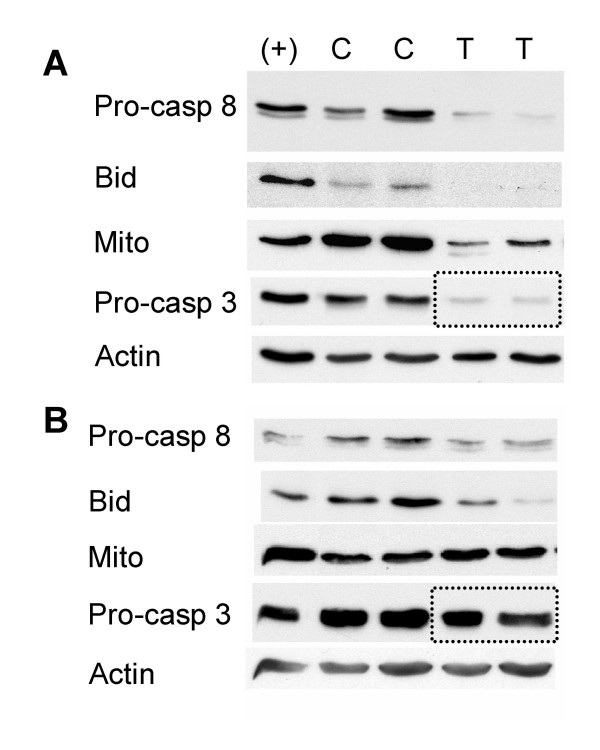
The effect of Apo2L/TRAIL on levels of procaspase 8, Bid, a marker of intact mitochondria, and procaspase 3 in Tumor #3 (Apo2L/TRAIL sensitive) and Tumor #4 (Apo2L/TRAIL resistant). Two tumors were removed from each group shown in Figure 5 on day 2 of treatment. The lanes are: (+) - positive control for each antibody; C- two tumors from the control group; T- two tumors from the Apo2L/TRAIL treated group; A. The Apo2L/TRAIL sensitive Tumor #3. By the second day of treatment with Apo2L/TRAIL, the levels of procaspase 8, Bid, the mitochondrial marker and procaspase 3 (outlined) in these tumors are greatly reduced. **B**. The Apo2L/TRAIL resistant Tumor #4. In the tumor that was resistant to Apo2L/TRAIL treatment, although there is a slightly diminished amount of procaspase 8 and Bid present in these tumors following 2 days of treatment with Apo2L/TRAIL, the levels of the mitochondrial marker and procaspase 3 (outlined) remain comparable to the controls. (In A and B a representative actin loading control is shown for each tumor).

To further investigate the response of a sensitive and resistant tumor to Apo2L/TRAIL, we analyzed tumors from the experiments with Tumors #3 and #4 shown in Figure 5. Two mice were removed from the control and Apo2L/TRAIL treated groups on the second day of treatment (6 hours following treatment) and these tumors analyzed by Western Blot (Figure 8). We observed a distinct difference in the levels of several critical molecules in the apoptotic pathways in response to Apo2L/TRAIL. In Tumor #3 (Figure 8A), which is sensitive to Apo2L/TRAIL, greatly reduced levels of procaspase 8 and Bid are detected in the tumors following Apo2L/TRAIL treatment. Reduced levels of intact human mitochondria are also observed and there is a clear reduction in detectable procaspase 3. Samples of Tumor #4 were similarly examined. As can be seen in Figure 8B, tumors treated with Apo2L/TRAIL have only slightly reduced amounts of procaspase 8 and Bid and the levels of intact mitochondria and procaspase 3 are comparable to those seen in untreated tumors. These results suggest that Tumor #3 undergoes activation of procaspase 8 and 3 in response to Apo2L/TRAIL and that signaling through Bid cleavage results in recruitment of the mitochondrial pathway and subsequently, apoptosis. On the other hand there is only partial activation of procaspase 8 in the resistant Tumor #4 suggesting that there is a defect upstream of caspase 8 activation which results in failure to adequately activate caspase 8 and thereby initiate apoptotic signaling. One possible explanation for this reduced activation of caspase 8 could be a defect in Apo2L/TRAIL receptor expression on the surface of these cells and this remains to be determined.

**Figure 9:**
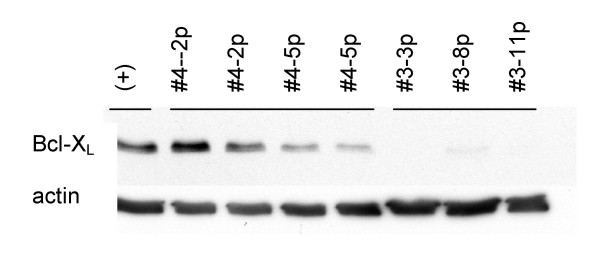
Comparison of Bcl-XL expression in a Tumor #4 that is resistant to Apo2L/TRAIL and Tumor #3 that is sensitive. Bcl-XL is detectable by Western blot in several passages of Tumor #4 (2nd passage, 5th passage) while Bcl-XL is not detectable in several passages of Tumor #3 (3rd passage, 8th passage, 11th passage).

It has been previously reported that resistance of established pancreatic cell lines to Apo2L/TRAIL and chemotherapy is in part associated with levels of the anti-apoptotic molecule Bcl-X_L _[35,43]. Therefore the expression of Bcl-X_L_, a molecule that can block the loss of mitochondrial membrane potential, was investigated in these tumors. Bcl-X_L _expression was found to be high in several samples of the resistant Tumor #4, compared to the sensitive Tumor #3 in which Bcl-X_L _was undetectable in several passages (Figure 9). This differential expression of the anti-apoptotic molecule Bcl-X_L _in a tumor that is resistant to Apo2L/TRAIL is consistent with the idea that in Tumor #4, in which only little caspase 8 activation occurs, Bcl-X_L _may play a role in the resistance by inhibiting activation of the intrinsic apoptotic pathway by the low levels of cleaved Bid.

Trauzold [37] characterized five pancreatic cell lines with regard to Apo2L/TRAIL sensitivity and concluded that although Bcl-X_L_was differentially expressed in sensitive (Capan1, Colo357) vs. resistant (PancTul, Panc89, Panc1) cells and made a significant contribution to the observed resistance to Apo2L/TRAIL, it is not the only factor. These authors concluded that resistance arose from the combined effects of the downregulation of pro-apoptotic molecules (FADD, Bid) and the concurrent upregulation of anti-apoptotic molecules (Bcl-X_L_, FLIP or IAP). Their experiments support the idea that there is a balance of several pro- and anti-apoptotic factors in pancreatic cells that ultimately determines the efficacy of the apoptotic signal. Recently, Bai et al. have found that knock-down of Bcl-XL in pancreatic cells that predominantly overexpress it, results in increased sensitivity of these cells to TRAIL in combination with other anti-tumor drugs [44]. The possible role of Bcl-X_L _and other critical molecules, particularly those upstream of caspase 8, in resistance of patient pancreatic tumors to Apo2L/TRAIL needs to be more fully investigated. Additionally, the mechanism(s) of the interaction between Apo2L/TRAIL and gemcitabine needs to be determined.

## Conclusion

Although much more work needs to be done, especially in evaluating a larger number of patients' tumors, these studies are important because they investigate for the first time the response of patient pancreatic tumors, grown as xenografts in SCID mice, to Apo2L/TRAIL. The results confirm previous work done with cell lines and support the idea that both the sensitivity and resistance to killing by Apo2L/TRAIL that has been observed in cell lines will be seen in patient tumors. Furthermore, these patients' tumors show heterogeneity of responsiveness both between tumors and within the same tumor that may be predictive of variability in a clinical setting. Importantly, it is encouraging that the combination of Apo2L/TRAIL with gemicitabine is able to enhance the antitumor efficacy and results in significant suppression of tumors that exhibit resistance to either one or both of these therapeutics. One potential benefit that remains to be explored is whether the enhanced efficacy achieved with combination therapy will allow lower doses of chemotherapy and/or Apo2L/TRAIL to be used, thus reducing the possibilities of toxicity and acquired resistance. The results of this study strongly support the further development of Apo2L/TRAIL as a therapeutic agent for the treatment of pancreatic adenocarcinoma.
